# Maternal–fetal microRNA axis in congenital heart disease: implications for tetralogy of Fallot

**DOI:** 10.3389/fcvm.2026.1821322

**Published:** 2026-04-28

**Authors:** Valeria Calcaterra, Clarissa Gervasoni, Chiara Ceriani, Savina Mannarino, Aurora Lanzotti, Filippo Puricelli, Irene Raso, Paolo Maugeri, Gloria Bertoli, Gianvincenzo Zuccotti

**Affiliations:** 1 Department of Internal Medicine, Pediatric and Adolescent Unit, University of Pavia, Pavia, Italy; 2 Pediatric Department, Buzzi Children’s Hospital, Milan, Italy; 3 Institute of Bioimaging and Complex Biological Systems, National Research Council (IBSBC-CNR), Segrate, Italy; 4 Pediatric Cardiology Unit, Buzzi Children’s Hospital, Milano, Italy; 5 Department of Biomedical and Clinical Science, University of Milano, Milano, Italy

**Keywords:** children, congenital heart disease, maternal–fetal, microRNA, pediatrics, tetralogy of Fallot

## Abstract

**Background:**

Tetralogy of Fallot (ToF), accounting for 7%–10% of congenital heart defects, represents the most prevalent cause of cyanotic CHD and is increasingly recognized as a lifelong condition characterized by progressive right ventricular (RV) dysfunction and heart failure.

**Objective:**

Since genetic variants explain only a minority of cases, this minireview highlights the role of microRNAs (miRNAs) as important epigenetic regulators in ToF pathogenesis.

**Methods:**

By integrating evidence from in silico bioinformatic pipelines, *in vitro* cellular validation, and *ex vivo* analyses of myocardial and circulating samples, we characterize a distinct miRNA signature that governs both early cardiac morphogenesis and postnatal remodeling in ToF.

**Results:**

Bioinformatic investigations have revealed extensive miRNome reprogramming, identifying master regulators such as miR-124, miR-222, and miR-1275 that converge on critical pathways involving inflammation, ferroptosis, and metabolic adaptation. Complementary, *in vitro* models have confirmed that miRNAs like miR-222 and miR-421 impair cardiomyocyte differentiation and trigger hypertrophic responses. These findings are further corroborated by *ex vivo* studies of human RV tissue and serum/plasma, where dysregulated miRNAs levels (i.e., miR-1, miR-133, and miR-21) correlate with clinical deterioration and structural maladaptation. Notably, a maternal–fetal miRNA axis emerges as a potential mediator between environmental factors and fetal development, shaping lifelong disease trajectories.

**Conclusions:**

Despite these advances, the causal relationships and temporal dynamics of miRNA regulation remain largely unresolved. Future integrative longitudinal studies and functional validations are essential to translate these epigenetic insights into novel biomarkers and targeted therapeutic interventions, ultimately improving long-term clinical outcomes in ToF patients.

## 1 Introduction

Tetralogy of Fallot (ToF) is a complex Congenital Heart Disease (CHD) characterized by conal septal misalignment with rightward aortic deviation, leading to a large subaortic ventricular septal defect, varying degrees of right ventricular (RV) outflow tract obstruction, and RV hypertrophy. Clinical presentation depends on obstruction severity and pulmonary blood flow reduction; newborns often present with cyanosis, while severe cases may develop hypoxemia and metabolic acidosis (1). As the most prevalent form of cyanotic CHD, ToF occurs in approximately 5–7 per 10,000 live births, accounting for about 7%–10% of all congenital cardiac malformations worldwide (2). Prenatal diagnosis is frequently achievable through fetal echocardiography, allowing optimized perinatal management (3). Surgical advances have raised long-term survival above 90%, but repaired patients often develop chronic pulmonary regurgitation and RV dysfunction, predisposing to arrhythmias, heart failure, and the need for lifelong follow-up and possible reinterventions (4, 5).

From an embryological perspective, ToF belongs to the spectrum of conotruncal defects arising from abnormalities in early cardiac morphogenesis. Human cardiogenesis occurs between the third and eighth week of gestation and requires tightly coordinated interactions among the first and second heart fields, cardiac neural crest cells, and endocardial cushions (6). Proper development of the outflow tract depends on the migration and contribution of neural crest–derived cells and second heart field progenitors to the conotruncal region. Disruption of these processes can lead to infundibular septal malalignment and abnormal truncus arteriosus septation, resulting in the characteristic anatomy of ToF (1, 7).

The etiology of ToF highlights limitations of a purely genetic model. Although high-throughput sequencing studies have identified pathogenic variants in cardiac developmental genes such as *TBX1*, *NKX2-5*, and *GATA4*, as well as chromosomal abnormalities such as 22q11.2 deletion syndrome, these account for only a minority of ToF cases (8–10). The high proportion of sporadic cases and the phenotypic discordance observed in monozygotic twins indicate that the genome alone cannot fully explain disease occurrence and variability. Such discordance likely reflects a complex interplay in which epigenetic modifications act as dynamic determinants, potentially mediating the effects of intrauterine environmental factors or interacting with stochastic genetic events, including post-zygotic mutations and somatic mosaicism. This reciprocal interplay between the epigenome and non-heritable factors suggests that ToF pathogenesis arises from multi-layered regulatory disruptions (11, 12).

**Figure 1:**
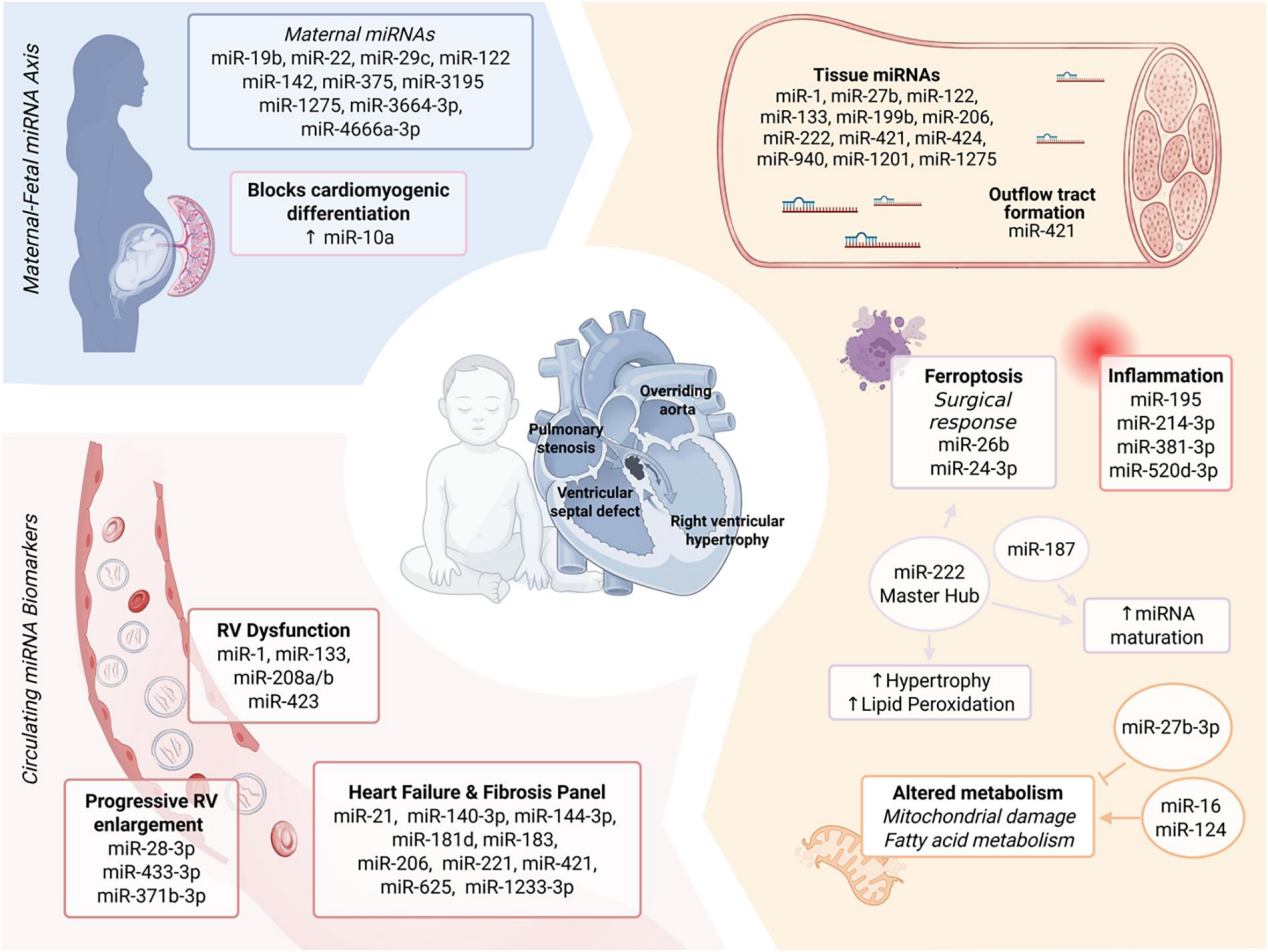
The microRNA landscape in tetralogy of Fallot: from maternal-fetal axis to postnatal remodeling. This schematic summarizes the integrated role of miRNAs across the ToF disease continuum. The top left illustrates the maternal–fetal axis, suggesting a potential influence of maternal miRNA imbalance on early cardiac development. The bottom left highlights circulating miRNA signatures associated with RV remodeling and disease progression. On the right, key cardiac tissue miRNAs are summarized, reflecting the complex molecular landscape underlying structural adaptation and long-term clinical evolution in ToF.

Cardiac development is therefore governed by a dynamic interplay of genetic, epigenetic, and environmental factors that converge to disrupt transcriptional regulation during critical developmental windows (Figure 1) (13, 14).

Among these epigenetic mechanisms, microRNAs (miRNAs) are small non-coding RNAs (∼22 nucleotides) that post-transcriptionally regulate gene expression (15). Over the past two decades, miRNAs have emerged as central regulators of cardiac development, orchestrating the spatial and temporal gene expression programs required for normal cardiogenesis. By fine-tuning pathways involved in cell proliferation, differentiation, migration, and apoptosis, miRNAs provide a flexible regulatory layer capable of integrating genetic background with environmental and developmental cues (16, 17). This regulatory capacity makes miRNAs particularly relevant in the context of complex CHD such as ToF, where subtle perturbations during early embryogenesis may translate into profound and lasting structural and functional consequences (18).

Beyond intrinsic genetic and epigenetic programs within the developing heart, increasing attention has been directed toward the influence of the intrauterine environment on cardiac morphogenesis. Maternal conditions such as metabolic dysregulation, hypoxia, inflammation, and nutritional imbalance are well-established exposome risk modifiers for CHD and are known to impact epigenetic regulation during early embryogenesis (19–21). Exposome refers to the sum of prenatal and periconceptional environmental exposures. Several factors have been associated with an increased risk of ToF, such as maternal hypoxia (due to high altitude, anemia, smoking) (22), air pollutants (23), exposure to heavy metals (arsenic, cadmium), drugs and teratogens, but also maternal diabetes and obesity, or oxidative stress and inflammation. In this setting, miRNAs are emerging as important mediators of maternal–fetal communication: they can be modulated by exposomal factors and circulate systemically within extracellular vesicles, crossing biological barriers such as the placenta.

Evidence from CHD cohorts supports the existence of a maternal–fetal miRNA axis, whereby altered maternal and placental miRNA profiles may influence fetal cardiac development during critical windows of susceptibility (24).

Although direct evidence linking this axis specifically to ToF is still limited, the biological plausibility is strong, given the sensitivity of outflow tract development and neural crest cell migration to tightly regulated gene expression. Viewing ToF within a maternal–child epigenetic continuum may help explain the high rate of sporadic cases, phenotypic variability, and long-term heterogeneity even after successful repair. In this context, miRNAs could act both as early modulators of disease and as accessible biomarkers connecting maternal, placental, and fetal compartments.

This minireview aims to synthesize current evidence on the role of miRNAs in the ToF pathogenesis, with particular focus on the proposed maternal–fetal miRNA axis and its potential implications for early cardiac development, postnatal RV remodeling, and long-term disease trajectory. The review also explores the potential of miRNAs as biomarkers and therapeutic targets across prenatal, fetal, and postnatal stages.

## 2 Methods

A non-systematic narrative literature search was conducted using major biomedical databases (PubMed and Scopus). Search terms included combinations of keywords such as “microRNAs”, “congenital heart disease”, “Tetralogy of Fallot”, “cardiac development”, and “maternal–fetal axis” and no time restrictions were applied.

Focusing on ToF, studies were included if they reported miRNAs differentially expressed in the disease compared with control groups or provided insights into the molecular mechanisms and etiopathogenesis. The selected studies were categorized according to the methodological approach used for miRNA evaluation and the type of biological samples or experimental models analyzed.

Additional literature was retrieved to contextualize and integrate the findings. The evidence was qualitatively synthesized, with a specific focus on the maternal–fetal miRNA axis and its potential role in ToF pathogenesis.

## 3 MicroRNAs in cardiac development and right ventricular remodeling

MiRNAs regulate cardiac development by controlling gene expression programs governing cardiomyocyte specification, proliferation, and differentiation. During embryogenesis, heart formation requires the precise temporal coordination of multiple signaling pathways, including Wnt/*β*-catenin, Notch, BMP, and TGF-*β*, all of which are targets of miRNA-mediated regulation (25, 26). Genetic ablation of the miRNA-processing machinery, such as Dicer or Drosha, causes severe cardiac malformations and embryonic lethality in animal models, underscoring the essential role of miRNAs in cardiogenesis (27, 28).

Several cardiac-enriched miRNAs have been identified as key regulators of early cardiac development. The miR-1/miR-133 cluster, among the most extensively studied myocardial miRNAs (myo-miRs), regulates cardiomyocyte proliferation and differentiation by targeting transcription factors and sarcomeric genes essential for myocardial maturation. Dysregulation of this cluster has been associated with abnormal ventricular development and conduction defects (29, 30). Similarly, miR-208 and miR-499, embedded within myosin heavy chain genes, modulate cardiomyocyte identity and contractile protein expression, thereby contributing to ventricular specification and postnatal cardiac adaptation (31–33). Beyond cardiomyocytes, miRNAs regulate non-myocyte populations, including cardiac fibroblasts and endothelial cells, thereby influencing extracellular matrix deposition and vascular development (34).

Of particular relevance to CHD, miRNAs are implicated in the development of the cardiac outflow tract, a process relying on the coordinated contribution of second heart field progenitors and cardiac neural crest cells. Specific miRNAs, including miR-17-92 cluster, miR-21, and members of the miR-196 family, have been shown to regulate neural crest cell migration, proliferation, and survival (35, 36). Since disruption of neural crest–dependent processes are central to the pathogenesis of conotruncal defects such as ToF, altered miRNA expression during early embryogenesis may represent a critical mechanism linking genetic susceptibility with aberrant cardiac morphogenesis. Beyond their developmental roles, miRNAs continue to shape cardiac structure and function throughout postnatal life by modulating adaptive and maladaptive remodeling responses (37). Under conditions characterized by chronic pressure or volume overload, miRNAs regulate cardiomyocyte hypertrophy, fibrosis, and apoptosis. In particular, the RV exhibits a distinct miRNA signature in response to increased afterload, reflecting its different embryological origin and molecular adaptation compared with the left ventricle. Experimental and clinical studies have reported differential expression of miRNAs such as miR-1, miR-208, and miR-133 in RV hypertrophy and failure, underscoring their role in regulating pathways involved in fibrosis, mitochondrial function, and cell survival (38–41).

In ToF patients, RV remodeling is a major determinant of long-term outcomes after surgical repair. ToF is defined by four classical anatomical hallmarks (ventricular septal defect, right ventricular outflow tract obstruction, overriding aorta, and right ventricular hypertrophy), which together impose chronic pressure overload and hypoxia on the myocardium. These structural anomalies not only drive mechanical stress but also establish a persistent molecular ‘developmental imprint’, whereby early embryonic signaling perturbations continue to influence myocardial adaptation. Persistent dysregulation of miRNAs, observed even long after anatomical correction, reflects this interplay between developmental and pathological processes, supporting the concept that ToF pathogenesis is shaped by a continuous epigenetic program from fetal life through adulthood.

## 4 Current evidence of microRNA dysregulation in tetralogy of Fallot

MiRNA dysregulation is increasingly recognized as a key epigenetic component in the onset and etiopathogenesis of ToF, involving both cardiac developmental processes and circulating compartments. Over the past decades, growing interest in miRNA biology has positioned these small non-coding RNAs within the broader framework of CHD research. A critical appraisal of the available evidence underscores the contribution of epigenetic mechanisms to disease initiation, progression, and clinical heterogeneity in ToF.

### 4.1 Bioinformatic evidence

Large-scale transcriptomic and bioinformatic investigations have substantially advanced the understanding of miRNA alterations in ToF, revealing a distinct epigenetic signature. Meta-analyses integrating multiple independent datasets consistently demonstrate extensive remodeling of the miRNome, with more than 200 dysregulated miRNAs converging on pathways related to cell cycle regulation, metabolic adaptation, and stress response (42). Within this systems-level context, network-based approaches have identified regulatory axes associated with specific pathogenic mechanisms. Inflammation-related signatures have highlighted miR-195-5p, miR-214-3p, miR-381-3p and miR-520d-3p as central nodes of a bioinformatic network model linked to immune activation, cytokine signaling, and stress-response pathways, supporting the concept that inflammatory programs are intrinsically embedded in the molecular landscape of ToF (43). Complementary integrative approaches have identified ferroptosis as an emerging pathogenic pathway, with miRNA–mRNA network reconstruction pointing miR-222 as a central hub connecting iron metabolism, lipid peroxidation, and cardiomyocyte susceptibility (44, 45).

Inclusion of noncoding RNA interactions has further refined regulatory network models. CircRNA-miRNA-mRNA interactome analyses identified miR-148a as a central mediator within regulatory axes implicated in cardiac morphogenesis and myocardial homeostasis (46). Metabolic dysfunction represents another recurring theme, as integrative studies implicating miR-16 and miR-124 in the regulation of mitochondrial activity and fatty acid metabolism, linking miRNA dysregulation to impaired energetic adaptation in the ToF myocardium (47). Consistent with the hemodynamic burden characteristic of ToF, RNA interactome analyses of RV hypertrophy under pressure overload revealed complex, age- and sex-dependent miRNA profiles, involving miR-216a, miR-371a, miR-372, and miR-5008 across distinct subgroups (48). Earlier miRNA–mRNA–transcription factor network studies further corroborate these findings, identifying miR-23b, miR-222, miR-93, miR-124, miR-138, miR-155, miR-187, miR-499 and miR-1275 as upstream regulators of widespread transcriptional remodeling in ToF (45, 49, 50). Collectively, these systems-level investigations indicate that ToF is characterized by coordinated perturbations of inflammatory, metabolic, and stress-response pathways linked to miRNA dysregulation, supporting the view of ToF as a disorder of network-level regulatory imbalance rather than isolated molecular defects.

Human myocardial datasets also provide evidence of dynamic stress-response mechanisms relevant to surgical intervention and disease progression. Gene-miRNA network analyses of atrial biopsy datasets reveal altered expression of ferroptosis-associated miRNAs, including miR-26b-5p and miR-24-3p, suggesting active modulation of oxidative and iron-dependent cell death pathways in pediatric ToF hearts (51).

### 4.2 *In vitro* validation

Experimental validation has confirmed several in silico predictions. Li et al. demonstrated differential expression of miR-187 and miR-222 identifying them as master regulators of cardiac miRNA maturation (45). Analyses in human RV tissue from aborted embryos, primary cardiomyocytes, and *in vivo* models showed that miR-222 promotes cardiomyocyte hypertrophy and increases lipid peroxidation markers. These effects were attenuated by co-expression of DICER1 or miR-133a, indicating that miR-222 impairs miRNA biogenesis through targeting of key processing components, including DICER1 and AGO2. These findings mechanistically link miR-222 dysregulation to structural remodeling and ferroptosis-associated stress pathways, supporting its role as a functional driver of ToF-like phenotypes. Earlier work by Zhang et al. (2013) further demonstrated that miR-222 enhances cardiomyocyte proliferation while impairing cardiomyocytes differentiation in primary embryonic mouse cardiomyocytes. In the same study, miR-424 upregulation promoted proliferation and inhibited migration, suppressing HAS2 and NF1, which were underexpressed in RVOT tissues of ToF patients, suggesting a potential role in abnormal cardiomyocyte behavior and right ventricular remodeling (52).

Additional cellular studies have elucidated the contribution of miR-421 to cardiac developmental signaling. Primary cardiomyocytes derived from the RV tissue of infants with ToF exhibit altered miR-421 expression compared with controls, with an inverse correlation between miR-421 and SOX4, a transcriptional regulator of the Notch pathway critical for outflow tract development (53). Moreover, in cardiomyocytes exposed to chronic hypoxia, miR-27b-3p attenuated apoptosis and mitochondrial damage through suppression of FOXO1 protein expression (54), suggesting a protective role under hypoxic stress conditions relevant to ToF pathophysiology.

### 4.3 *Ex vivo* analyses of myocardial and circulating samples

Comparative analyses of myocardial tissue from infants with non-syndromic ToF and controls have consistently demonstrated distinct miRNA expression patterns. RT-qPCR validation identified 18 significantly dysregulated miRNAs in RV samples (52).

Across independent tissue-based studies, a core set of miRNAs, including miR-1, miR-27b, miR-122, miR-133, miR-199b-5p, miR-206, miR-222, miR-421, miR-424, and miR-940, miR-1201, miR-1275 have emerged. The altered expression patterns of these miRNAs reflect developmental perturbations with postnatal remodeling and responses to surgical stress (45, 53, 55–61).

Beyond myocardial tissue, circulating miRNAs have been investigated as minimally invasive biomarkers in pediatric and adult ToF cohorts. Circulating levels of miR-1, miR-133a, miR-208a/b, and miR-423-5p correlate with RV dilation and systolic dysfunction in repaired ToF patients. Progressive RV enlargement has been associated with increasing levels of miR-28-3p, miR-433-3p, and miR-371b-3p (62, 63). Additional panels identified in pediatric and mixed-age cohorts, including miR-21-5p, miR-140-3p, miR-144-3p, miR-181d-5p, miR-183-5p, miR-206, miR-221-5p, miR-421, miR-625-5p, and miR-1233-3p, have been associated with heart failure progression, myocardial fibrosis, and clinical deterioration (64–66).

The miRNAs consistently associated with ToF in at least two independent studies are summarized in Table 1. For each entry, it was highlighted the analytical approach, the biological samples, the experimental techniques employed, and the direction of dysregulation (up- or down-regulation) as described in the original studies.

**Table 1:** Summary of miRNAs involved in tetralogy of Fallot.

miRNA	Approach	Sample/model	Method	Direction	References
miR-1	*ex vivo*	Serum	RT-qPCR	DE	(62)
		RV myocardial tissue	RT-qPCR; Sequencing	↓; DE	(58,59)
miR-21	*ex vivo*	Plasma	RT-qPCR	↓	(64)
		Serum		↑	(66)
		RV myocardial tissue	microarray	↑	(52)
miR-124	*in silico*	GEO database	Microarray	↓	(47,49)
miR-133	*ex vivo*	RV myocardial tissue	Sequencing	DE; ↓	(55,58)
	*ex vivo*	Serum	RT-qPCR	DE	(62)
miR-206	*ex vivo*	RV myocardial tissue	RT-qPCR	↓	(59)
		Venous blood from cubital vein	Microarray, validation RT-qPCR	↓	(65)
miR-222	*in silico*	GEO database	Microarray, RNA-sequencing	↑	(50)
	*in silico, ex vivo*	GEO database, cardiac tissue	Sequencing	DE	(44)
	*ex vivo, in vitro*	RV myocardial tissue, mouse cell line	Microarray, RT-qPCR	↑	(52)
	*in silico, in vivo, in vitro, ex vivo*	GEO database, RV tissue, hPCM, mouse model	RT-qPCR	↑	(45)
miR-421	*ex vivo*	Venous blood from cubital vein	Microarray, RT-qPCR	↓	(65)
	*ex vivo*	RV myocardial tissue		↑	(57)
	*ex vivo, in vitro*	RV tissue, hPCM	RT-qPCR	↑	(53)
	*in silico, ex vivo, in vitro*			↑	(61)
miR-1275	*in silico*	GEO database	Microarray, RNA-sequencing	↑	(50)
	*ex vivo*	RV myocardial tissue	Microarray, RT-qPCR	↑	(57)

RV, right ventricular; hPCM, human primary cardiomyocytes; DE, “differentially expressed” with not defined directionality.

## 5 Maternal–fetal microRNA axis in congenital heart disease and implications for tetralogy of Fallot

Circulating maternal miRNAs could represent effective molecular indicators of maternal-fetal interactions and, under pathological conditions, can reflect fetal cardiac abnormalities rather than physiological pregnancy alone. Many pregnancy-associated miRNAs originate from the placenta and are selectively released into maternal circulation, showing tightly regulated expression patterns linked to fetal development (67, 68). In the context of CHD, altered maternal miRNA profiles have been proposed as markers of perturbed fetal cardiogenesis, implicating miRNA-mediated signaling in early cardiac morphogenesis (69).

Jin et al. identified exosomal miR-199a-3p and miR-146a-5p as potential non-invasive biomarkers for the early detection of fetal ventricular septal defects (VSDs), as both were significantly downregulated in maternal serum (70). Similarly, decreased miR-99a expression in maternal blood correlated with fetuses affected by CHD, indicating a role in fetal development (71).

Evidence from mixed CHD cohorts shows that maternal circulating miRNAs, including miR-19b, miR-22, miR-29c, miR-142-5p, miR-375, miR-1275, miR3664-3p, and miR-4666a-3p are upregulated, whereas miR-122-5p and miR-3195, are downregulated in pregnancies carrying affected fetuses, compared with controls (24, 69, 72).

These findings suggest that early cardiac morphogenesis can be modulated by maternal-fetal microRNA communication. The rapid normalization of these alterations within 24 h after delivery underscores their tight association with fetal pathology and supports their value as pregnancy-specific biomarkers of abnormal cardiac development (24). Although these studies do not distinguish among CHD subtypes, they outline a mechanistic framework whereby maternal miRNA signatures may influence early cardiac development.

Downregulation of miR-1, miR-208, and miR-499 has been observed in umbilical cord blood of subjects with CHD fetuses, suggesting their potential use in prenatal screening for CHD (73).

Despite strong biological plausibility, evidence specifically linking the maternal–fetal microRNA axis to ToF remains limited. A recent analysis of exosomal miRNAs in amniotic fluid revealed significant dysregulation of miRNAs involved in cardiac development and validated their functional role in cardiomyocyte differentiation. In particular, miR-10a-5p overexpression demonstrated an inhibition in cardiomyogenic differentiation by directly suppressing TBX5 expression, which is a known cardiomyocyte marker gene (74). These findings provide initial proof that miRNA perturbations are detectable in fetal-derived compartments during gestation and may precede postnatal manifestations of RV remodeling.

The overlap between prenatal miRNAs involved in cardiac development and those persistently dysregulated in postnatal ToF myocardium supports a model in which early maternal-fetal miRNA perturbations contribute to long-term cardiac disease programming. Within this framework, miRNAs may act not only as non-invasive biomarkers of early cardiac maldevelopment but also as molecular mediators linking intrauterine conditions to lifelong structural and functional cardiac outcomes in ToF.

## 6 Knowledge gaps and future perspectives

Despite significant advances in characterizing miRNA dysregulation in ToF, three critical gaps remain: the reliance on postnatal samples, which obscures the distinction between primary pathogenic drivers and secondary adaptive responses like RV remodeling; the lack of systematic, longitudinal investigation into the maternal-fetal miRNA axis; and the absence of multi-compartment analyses to explain how early epigenetic perturbations translate into lifelong clinical phenotypes. Future research must employ integrated multi-omic approaches and functional models to validate the role of the exposome in modulating these pathways. By identifying conserved miRNA signatures across developmental stages, it will be possible to reconceptualize ToF from a static congenital defect into a dynamic, lifelong condition shaped by the continuous interplay of genetic, epigenetic, and environmental factors (75).

## 7 Discussion

ToF represents not only a structural congenital defect but also a lifelong condition shaped by dynamic interactions between genetic, epigenetic, and environmental factors. MiRNAs emerge as central regulators linking early cardiac development, postnatal RV remodeling, and maternal–fetal influences. Distinct miRNA signatures in cardiac tissue, circulating profiles, and emerging prenatal studies implicate pathways in cardiomyocyte differentiation, outflow tract formation, fibrosis, and metabolic adaptation. While causal relationships and mechanistic roles remain incompletely understood, understanding miRNA regulation in ToF holds promise for novel biomarkers, early detection strategies, and targeted interventions to improve lifelong outcomes.
